# A Zero Velocity Detection Algorithm Using Inertial Sensors for Pedestrian Navigation Systems

**DOI:** 10.3390/s101009163

**Published:** 2010-10-13

**Authors:** Sang Kyeong Park, Young Soo Suh

**Affiliations:** Department of Electrical Engineering, University of Ulsan, Namgu, Ulsan 680-749, Korea; E-Mail: damiro76@hotmail.com

**Keywords:** hidden Markov model, pedestrian navigation system, zero velocity update method, Kalman filter

## Abstract

In pedestrian navigation systems, the position of a pedestrian is computed using an inertial navigation algorithm. In the algorithm, the zero velocity updating plays an important role, where zero velocity intervals are detected and the velocity error is reset. To use the zero velocity updating, it is necessary to detect zero velocity intervals reliably. A new zero detection algorithm is proposed in the paper, where only one gyroscope value is used. A Markov model is constructed using segmentation of gyroscope outputs instead of using gyroscope outputs directly, which makes the zero velocity detection more reliable.

## 1. Introduction

A pedestrian or personal navigation system provides position information about a person indoors or outdoors. For example, if a firefighter carrying a personal navigation system is injured in the line of duty, we can know her/his position from the information transmitted by a pedestrian navigation system. Pedestrian navigation systems using inertial sensors are proposed in [1–6]. The main advantage of inertial sensor-based systems is that they are environment-independent; pedestrian navigation systems using vision or wireless communication require that landmarks or wireless nodes be installed in the environments [7,8].

There are many different pedestrian navigation systems using inertial sensors. They use similar inertial navigation algorithms to compute the position [9,10]. Also they all use zero velocity updating algorithms. When an inertial navigation algorithm is used, the position and velocity errors diverge by a few seconds without error resetting. During normal walking cycles, a foot touches the ground almost periodically and stays on the ground for a short time (usually about 0.1∼0.3 s), which is called the zero velocity interval. In the zero velocity updating algorithm, this zero velocity interval is detected and thus the velocity error is reset to zero. Accordingly the position and velocity errors diverge very slowly, and consequently being able to reliably detect this zero velocity interval is an important issue.

**Figure 1.**
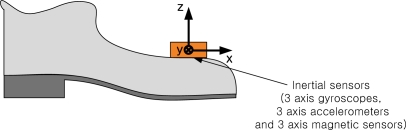
Inertial sensors for the pedestrian navigation system.

Slightly different zero detection algorithms are used in [1–3,5,6]. In [1], the zero velocity interval is determined based on gyroscope and accelerometer output norms. If the outputs are less than some threshold for a predetermined time, the zero velocity interval is decided. In [2,3], similar algorithms are used, where the main difference is which sensor values are used. In [2], the zero velocity interval is determined based on *z*-axis accelerometer and *y*-axis gyroscope outputs (see Figure 1 for the coordinate frame). In [3], the zero velocity interval is determined based on norms of gyroscope outputs. In [5], the zero velocity interval is detected based on the variance of accelerometer values. In [6], the zero velocity interval is detected based on norms of accelerometers and gyroscopes along with variance of accelerometers.

To use the zero velocity algorithms in [1–3,5,6], some threshold values (both for sensor values and time duration) must be assigned. These threshold values could differ significantly when a person is walking and running. The sensor values tend to become large and the interval duration tend to become shorter when a person is running. If we choose the threshold values too small, then we cannot detect zero velocity intervals when a person is running. On the other hand, if the threshold values are too large, then we could detect wrong zero velocity intervals. In general, the methods in [1–3,5,6] are relatively good for walking scenarios, but not for both walking and running.

In this paper, we propose a new zero velocity detection algorithm using a hidden Markov model [11,12]. The most relevant result is [4], where a zero velocity detection algorithm using a hidden Markov model is also used. The main differences are twofold. One is that only one gyroscope value is used in this paper while a foot sensor is also used in [4], which makes the zero detection problem relatively easy. The other difference is that a Markov model is constructed using segmentation of gyroscope outputs instead of using gyroscope outputs directly, which makes the zero velocity detection more reliable.

## 2. Inertial Sensor-Based Pedestrian Navigation Systems and Zero Velocity Intervals

In inertial sensor-based pedestrian navigation systems, inertial sensors are installed on a shoe, as can be seen in Figure 1. Note that the *y*-axis is nearly perpendicular to the sagittal plane. Thus, when a person walks or runs, the dominant rotation axis is the *y*-axis. We use *y*-axis gyroscope value to determine the zero velocity intervals. The gyroscope measures angular rate of foot rotations, and *y*-axis gyroscope output in our system has positive values when a foot is rotating clockwise.

**Figure 2.**
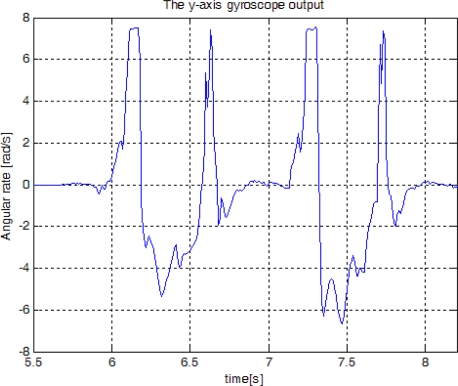
The y-axis gyroscope output in walking.

**Figure 3.**
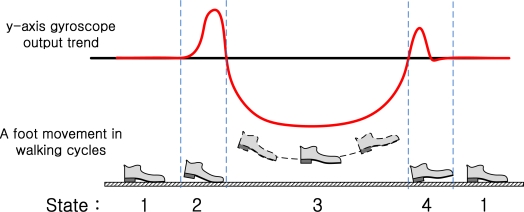
*y*-axis gyroscope value trend and a foot movement in normal walking cycles.

Figure 2 shows the *y*-axis gyroscope output of two walking steps. From experiments, we found that a typical pattern is given as in Figure 3, where the pattern consists of four segments. The pattern starts with the zero value segment and it has two positive value segments and one negative value segment between them. These four segments are related to the foot movement. When a foot is on the ground, the output value is near upon zero. As a foot takes off the ground, the gyroscope output has positive values. And then it has negative values when a foot is swinging. Lastly, gyroscope output has positive values once more when the heel of a foot (or shoe) contacts the ground. This pattern is repeated in walking and running. We assign four states (1,2,3, and 4) to each segment, as in Figure 3. The details are discussed in Section 3.

Figure 3 shows how the *y*-axis gyroscope value changes during the normal walking cycles. A foot touches the ground almost periodically for a short interval. During this short interval, a foot is fixed on the ground and not moving. These short intervals are called “zero velocity intervals”. Due to sensor noises in the real data, it is not always easy to determine the zero velocity interval. Similar patterns can be observed during running cycles. In the running cycles, the zero velocity interval becomes shorter and it is more difficult to detect the zero velocity interval. We divided the gait pattern into four states based on the features of the y-axis gyroscope output. At this time, the state 1 is zero velocity interval.

## 3. Hidden Markov Model

**Figure 4.**
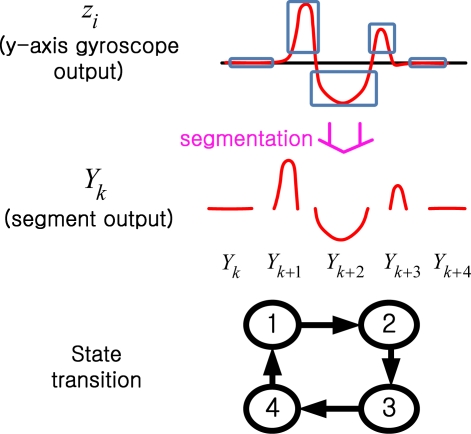
Hidden Markov model based on segmentation of *z_i_*.

In this section, we introduce a hidden Markov model for the zero velocity detection. The walking states are modeled as a finite state machine (see Figure 4), whose states can be observed through *y*-axis gyroscope value *z_i_* Instead of using *z_i_* directly as in [4], *Y_k_* (a series of segments derived from *z_i_*) is used as an output in the hidden Markov model.

**Figure 5.**
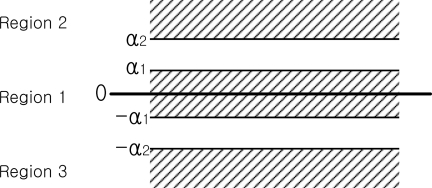
The region classification.

The segmentation of *y*-axis gyroscope value *z_i_* is explained. First three regions (Region 1, 2, and 3) are defined depending on *z_i_* values (see Figure 5). To formally state this, we define a function *f*(*z_i_*):

(1) $$f(z_{i})=\{\begin{array}{cc} 1, & |z_{i}|\leq \alpha_{1}\\ 2, & z_{i}>\alpha_{2}\\ 3, & z_{i}<-\alpha_{2}\\ 0, & \text{otherwise} \end{array}\text{where}\;0<\alpha_{1}\leq \alpha_{2}\;.$$

If *z_i_* values stay in the region *j* more than *N_j_* (*j* = 1,2,3) (that is, at least *N_j_* consecutive *z_i_* values have the same *f*(*z_i_*)), those *z_i_* values make a segment.

**Figure 6.**
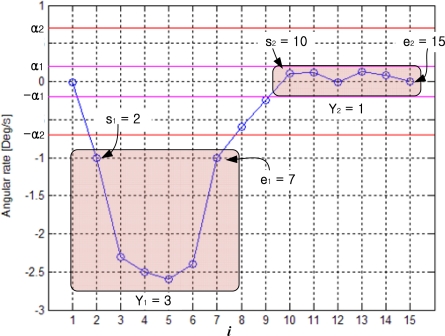
Example of a segmentation.

A segmentation example of is given in Figure 6 with *N*_1_=*N*_2_=*N*_3_=3. Since six consecutive *z_i_* (2≤*i*≤7) values have the same *f*(*z_i_*) =3 (that is 6≥ *N*_3_ = 3), they form the first segment. We will use *Y*_k_ (*k* is the segment index) to denote the segment value, which is defined by *Y*_k_ =*f*(*z_i_*), where *z_i_* belongs to the *k*-th segment. In Figure 6, we note that *Y*_1_=3. Similarly, the second segment is *Y*_2_=1. Also note that some *z_i_* values do not belong to any segments. For example, *z*_8_ and *z*_9_ do not belong to any segments and these values are ignored.

In Figure 6, *s_k_* and *e_k_* denote the starting and ending indices of the *k*-th segment, respectively: note that *s*_1_ = 2, *e*_1_ = 7, *s*_2_ =10 and *e*_2_ =15.

**Figure 7.**
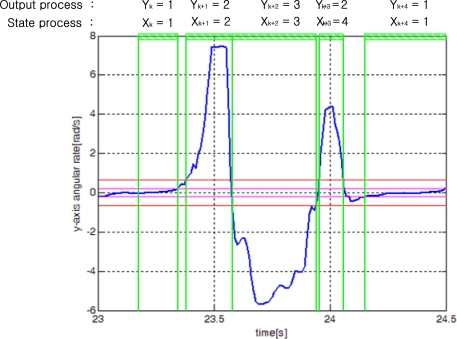
State transition example of normal walking cycles.

We will identify each segment using the finite state hidden Markov model. We assume that there is a Markov process *X_k_*, which represents four walking states in Figure 3. *X_k_* assumes one of four states: 1, 2, 3, and 4. When a person is walking, a typical state transition is 1→2→3→4→1→…. The Markov process *X_k_* cannot be obtained directly but is estimated through the output *Y_k_* (that is, segment output), which can be observed. An example of state transitions of *X_k_* and output *Y_k_* is given in Figure 7.

**Figure 8.**
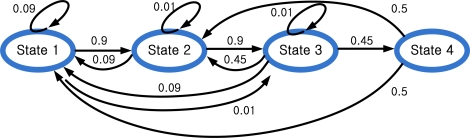
The state transition diagram.

From experimental results, we determined the state transition probability as in Figure 8. A typical state transition is 1→2→3→4→1. However, there are also other possible transition patterns. For example, 4→3→2 transition (state 1 is missing) is possible. In this case, output data corresponding to state 1 is too short to be recognized as *Y_k_* and state 1 is considered to be missing.

From Figure 8, the state transition probability matrix *A* is given by:

(2) $$A=\left[\begin{array}{cccc} 0.09 & 0.09 & 0.09 & 0.5\\ 0.9 & 0.01 & 0.45 & 0.5\\ 0.01 & 0.9 & 0.01 & 0\\ 0 & 0 & 0.45 & 0 \end{array}\right]$$

where the (*m,n*) -th element of *A* is given by:

$$A_{\mathrm{mn}}=\text{Pr}[X_{k+1}=m|X_{k}=n],\;\;\;\sum_{m=1}^{4}A_{mn}=1.$$

The determination of *A* is a trial-and-error process, which is explained in the following. A normal state transition is 1→2→3→4→1→2→3→4. If walking and running cycles consist of only normal transitions, *A* should be as follows:

$$A=\left[\begin{array}{cccc} 0 & 0 & 0 & 1\\ 1 & 0 & 0 & 0\\ 0 & 1 & 0 & 0\\ 0 & 0 & 1 & 0 \end{array}\right]$$

However, we found that some states are missing during walking and running. A person walked or ran 30 steps and we examined *Y_k_*. If no state is missing, *Y_k_* should be 1→2→3→2 for one walking or running cycle. If *Y_k_* is 1→2→3→1, then we know state 4 is missing. The results are summarized in Table 1.

From Table 1, we can draw the following conclusions:

- State 1 is sometimes missing (in particular, when a person is running)
- State 2 and 3 are not missing
- State 4 is sometimes missing (in particular, when a person is running).

Thus we modified *A*_1_ to allow the following state transitions: 1→2→3→1 (state 4 is missing), 1→2→3→4→2 (state 1 is missing), and 1→2→3→2 (state 1 and 4 are missing):

$$A_{1}=\left[\begin{array}{cccc} 0 & 0 & 0 & p_{3}\\ 1 & 0 & p_{1} & p_{4}\\ 0 & 1 & 0 & 0\\ 0 & 0 & p_{2} & 0 \end{array}\right]$$

If we choose large *p*_1_ (for example *p*_1_= 0.9 and *p*_2_= 0.1), it tends to estimate the transition 1→2→3→4 (normal cycle) as 1→2→3→2 (state 1 and 4 are missing). On the other hand, if we choose small *p*_1_ (for example, *p*_1_ = 0.1 and *p*_2_ = 0.9), it tends to estimate the transition 1→2→3→2 (state 1 and 4 are missing) as 1→2→3→4 (normal cycle). If difference between *p*_1_ and *p*_2_ is not large, the estimation results are similar.

Finally, we added small values for most transitions to allow unusual transitions. For example, when a person is standing and idly moving foot, unusual transitions (such as 2→1) could happen.

We have explained guidelines of how *A* are determined. Some values (such as 0.01 and 0.09) are determined by changing values and testing the results. We note that the proposed *A* is by no means an optimal *A*. As far as we know there is no analytic design procedure to derive the optimal *A* as stated in [13]. It is possible that extensive tuning of the parameters could produce better results and to derive a better tuning method is one of future topics.

If the output *Y_k_*=1, then we can assume *X_k_*=1 from Figure 7. Also if *Y_K_*=2 then *X_k_* is either 2 or 4. If *Y_k_*= 3, then *X_k_* is 3. This observation is contained in a matrix *C*, which represents the link between the output process *Y_k_* and the state process *X_k_*. Matrix *C* is defined by:

$$C_{\mathrm{mn}}=\text{Pr}[Y_{k}=m|X_{k}=n],\;\;\sum_{m=1}^{3}C_{\mathrm{mn}}=1.$$

In this paper, we use the following *C* matrix:

(3) $$C=\left[\begin{array}{cccc} 1 & 0 & 0 & 0\\ 0 & 1 & 0 & 1\\ 0 & 0 & 1 & 0 \end{array}\right]$$

Once *A* and *C* are defined, we can estimate *X_k_* from the output *Y_k_* using HMM filters or smoothers [11–14]. Let Π*_k|k+l_* ∈*R*^4×1^ be a probability vector whose *i*-th element is given by Pr[X*_k_*=*i|Y*_1,_⋯,*Y_k+l_*]. If *l=*0, then the problem becomes an HMM filter. If *l*>0, the problem becomes an HMM (fixed-lag) smoother problem. It is known that an HMM smoother gives a more reliable result than an HMM filter [11]. We found an HMM smoother with *l=*1 gives a significantly much more reliable results in our case (see the experimental results in Table 1). For example, suppose the state 4 and 1 are missing and the state transitions are given by 1→2→3→2→3 (*X_k_*). In the case, the outputs are given by 1→2→3→2→3(*Y_k_*). If we use an HMM filter, the estimated first four states are likely to be 1→2→3→4 (*X_k_*) since it only uses the current outputs 1→2→3→2(*Y_k_*). On the other hand, an HMM smoother gives the correct estimates using the outputs 1→2→3→2→3(*Y_k_*). When an HMM smoother with *l=*1, we can obtain an estimation result only after the current and the next outputs are available. Thus the estimation result is delayed by the combined duration of the current and next segments: typical delay is 1 second.

## 4. Zero Velocity Detection Algorithm

After *X_k_* is estimated, the next step is to determine zero velocity intervals. We decide that the time *i* belongs to zero velocity intervals if |*z_i_*|≤α_3_ with anti-chattering algorithm and *i* satisfies the following conditions:

$$\begin{array}{ll} \text{case}\;1:\;\text{if} & X_{k-1}=1,X_{k}=2\\ & s_{k-1}+\beta_{1}\leq i\leq e_{k-1}-\gamma_{1}\\ \text{case}\;2:\;\text{if} & X_{k-1}=4,X_{k}=2\\ & s_{k-1}+\beta_{2}\leq i\leq s_{k}-\gamma_{2}\\ \text{case }\;3:\;\text{if} & X_{k-1}=3,X_{k}=2\\ & s_{k-1}+\beta_{3}\leq i\leq s_{k}-\gamma_{3}. \end{array}$$

The |*z_i_*|≤α_3_ condition is used as a safeguard to prevent false identification of the zero velocity interval.

Case 1 corresponds to the normal walking cycles, where the state transition is 3→4→1→2 (see Figure 7). Since the zero velocity interval belongs to the state 1, the interval is chosen inside state 1. The parameters *β*_1_ and *γ*_1_ are used to discard time intervals near edges of the interval whose state is 1:

$$\beta_{1}=\gamma_{1}=0.1(e_{k-1}-s_{k-1})\;.$$

Since a foot is moving before and after state 1, the foot velocity cannot be zero in the edge regions. Thus we cut 10% (corresponding to 0.1) off the interval at both edges. If we choose larger values (for example, 0.2), the chance that a non-zero velocity interval is mistakenly identified as a zero velocity interval is reduced. However, we could obtain narrower zero velocity intervals, which is not desirable.

**Figure 9.**
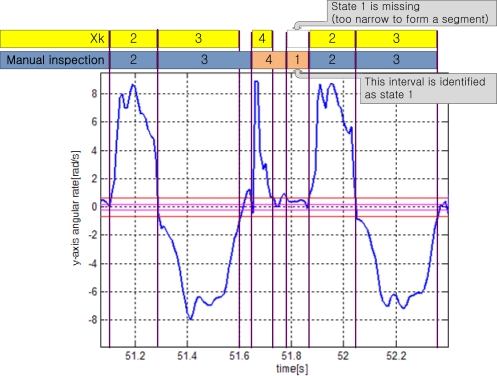
Case 2: state 1 is missing.

Case 2 and 3 correspond to walking or running cycles, where state 1 and/or state 4 are missing. Case 2 is when state 1 is missing. This could happen if the zero velocity interval is too short to form a segment. For example, state 1 is missing in Figure 9: state transition is 2→3→4→2→3. However, we can identify an interval corresponding to state 1 in Figure 9 by the manual inspection. This interval is identified as state 1 in the manual inspection. Thus the state classifications by the manual inspection and by *X_k_* are different: even if some states are missing in *X_k_* transition, they can be identified in the manual inspection.

We manually inspected the data and found the average duration ratio of each state is given as follows:

(state 1,  state 2,  state 3,  state 4)=(11.3%, 27.4%, 42.8%, 18.5%).

The ratio was computed from a weighted average of walking (weighting 0.1) and running (weighting 0.9) data. More weighting was given to running since missing states (like in case 2 and 3) are more likely to happen in running.

**Figure 10.**
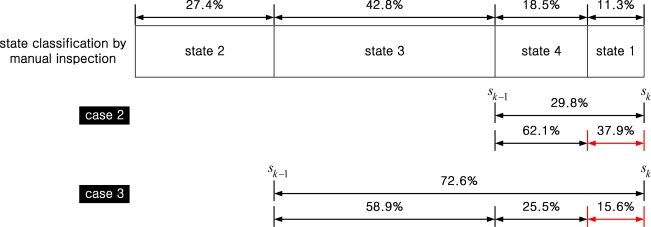
Average duration ratio of each state by manual inspection and its use in Case 2 and 3.

In case 2, the state 1 is missing. We assume the interval of missing state 1 is (*s_k_*_–1_+*b_k_,s_k_*). Note that the average duration percentage of state 4 and state 1 are 18.5% and 11.3%, respectively. Thus the duration ratio between *s_k_*_–1_ and *s_k_* is 0.621:0.379. Thus we choose *β*_2_ and *γ*_2_ as follows (see Figure 10):

$$\beta_{2}=0.621(s_{k}-s_{k-1}),\;\;\;\gamma_{2}=0$$

In other words, the interval [*s_k_*_–1_, *s_k_*] is divided into [*s_k_*_−1_,*s_k_*_−1_+*β*_2_] and [*s_k_*_−1_ +*β*_2_,*s_k_*], where the latter is considered as state 1 interval.

Case 3 is when state 4 and 1 are missing. In this case, interval [*s_k_*_–1_, *s_k_*] includes the state 3 and missing states (4 and 1). Note that the average duration percentage of state 3, 4, and state 1 are 42.8%, 18.5%, and 11.3%, respectively. Thus the duration ratio among state 3:state 4:state 1 is 0.589 : 0.255 : 0.156. Thus we choose *β*_3_ and *γ*_3_ as follows:

$$\beta_{3}=0.844(s_{k}-s_{k-1}),\;\;\gamma_{3}=0$$

**Figure 11.**
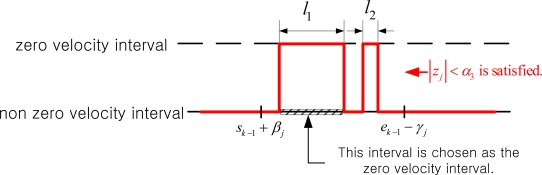
Zero velocity interval selection.

When the interval [*s_k_*_–1_+*β_j_*, *e_k_*_–1_−*γ_j_*] is determined, we checked whether *z_i_*, *i*∈[*s_k_*_–1_+*β_j_*, *e_k_*_–1_−*γ_j_*] satisfies |*z_i_|*<*α_3_* condition. Let *U* = {*i*| |*z_i_*| < α_3_, *i* ∈ [*s*_*k*−1_ + β_*j*_,*e*_*k*−1_−γ_*j*_]}. If the set *U* consists of more than one intervals as in Figure 11, the interval whose duration is the largest is chosen as the zero velocity interval.

## 5. Experiments

**Figure 12.**
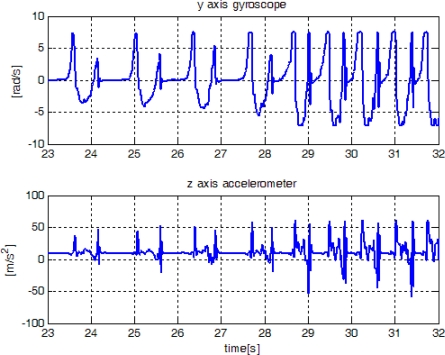
Sensor outputs when a person is walking(23∼27 s) and slowly running(27∼32 s).

In this section, the proposed method is verified using experiment data, which are obtained from an inertial sensor unit (Xsens Mti) attached on a shoe. The sampling rate is 100 Hz. In Figure 12, sensor outputs are given when a person walked and then ran slowly. We note that only gyroscope output is used in the proposed algorithm. The z-axis accelerometer output is also given since it is used by other existing algorithms. The proposed algorithm along with other existing algorithms [1–3] are applied to the experiment data. The parameters of the proposed algorithm are given by:

$$\alpha_{1}=\alpha_{2}=\alpha_{3}=0.7,N_{1}=N_{2}=10,N_{3}=20,l=1$$

The parameters *α_j_* and *N_j_* are determined from the experimental data so that normal walking gives four different states. For example, if we use a smaller *α*_2_ value, it becomes sensitive to noises so that states 2/3/4 could be falsely identified. On the other hand, if we use larger *α*_2_ value, states 2/3/4 could be lost. The parameter *N_j_* is closely related to *α_j_*. For example, smaller *N*_2_ has a similar effect of using smaller *α*_2_ in the sense that state 2/3/4 could be falsely identified.

As a reference, zero velocity interval is determined by manually analyzing gyroscope data. When data are analyzed manually from the plot, the sensor noises and irregularities can be easily spotted and thus we can consider the detected interval as a true zero velocity interval.

**Figure 13.**
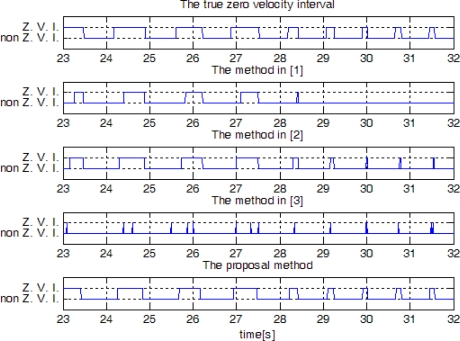
Zero velocity detection comparison when a person is walking and slowly running (Z. V. I. means zero velocity interval).

The result is given in Figure 13. The algorithm in [1] failed to detect the zero velocity interval when a person is running. The algorithm in [3] is not reliable in the sense that there are false zero velocity detections.

**Figure 14.**
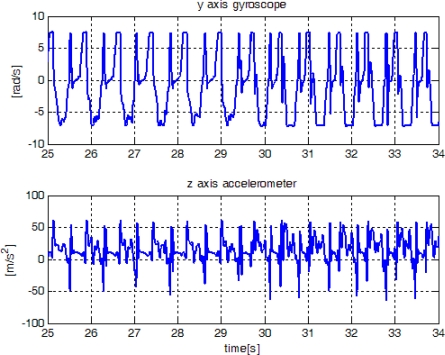
Sensor outputs when a person is running slowly(25∼30s) and running fast(30∼34 s).

**Figure 15.**
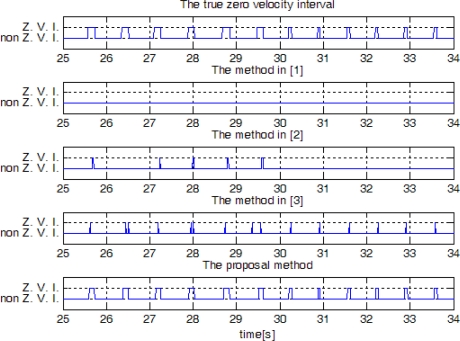
Zero velocity detection comparison when a person is running slowly and running fast(Z. V. I. means zero velocity interval).

In Figure 14, sensor outputs are given when a person ran slowly and then ran fast. The speeds of “walk”, “run slowly” and “run fast” are about 1.05 m/s, 2.14 m/s, and 3.50 m/s, respectively, which are computed by dividing the distance by the elapsed time. The zero detection results are given in Figure 15. It can be seen that the methods in [1] and [2] cannot detect the zero velocity intervals when a person ran fast. In all cases (Figures 13 and 15), the proposed algorithm reliably detected the zero velocity intervals.

To compare performance of each method quantitatively, a person walked or ran 30 steps at different speed. Since there is one zero velocity interval in each step, 30 zero velocity intervals should be detected. The number of detected zero velocity intervals by different methods is given in Table 2. We can see when a person is walking, all methods give a good result. When a person is running, method 1 and 2 failed to detect the zero velocity intervals most times. The method [3] and the proposed method with an HMM smoother are able to detect all the zero velocity intervals. When counting the number of detected zero velocity intervals, we counted one even if there are more than two detection during the same zero velocity interval. For example, see the two detected zero velocity intervals by the method [3] between 24 and 25 s. These two intervals are counted as one interval. We note that there is a zero velocity interval between the two detected zero velocity intervals, but it is not detected by the method [3]. On the other hand, the proposed method detects almost the whole range of a zero velocity interval. Thus the proposed method can detect the zero velocity intervals more reliably.

We also investigated whether there is false zero velocity detection (an interval is identified as a zero velocity interval while a foot is not in the zero velocity interval). The result is given in Table 3, where the same experiment data were used as in Table 2. It can be seen that the method in [3] sometimes falsely identifies the zero velocity intervals: we found the method in [3] sometimes falsely identifies zero crossing points of gyroscope norm as zero velocity intervals. There was no false zero velocity detection in other methods.

## 6. Conclusions

In this paper, we have proposed a new zero velocity detection algorithm, which can be used in inertial sensor based pedestrian navigation systems.

Existing zero detection methods usually work well for normal walking cycles, but the detection is not reliable for running cycles, in particular when the running speed is high. Using a hidden Markov model, the proposed algorithm can analyze both walking and running cycles and the zero velocity interval is thus more reliably detected.

The actual state transition probabilities differ depending on speed of a person. In this paper, we used one averaged transition probability. It is a future work to derive a zero velocity detection algorithm that uses multiple state transition probability matrices.

## Figures and Tables

**Table 1.** Missing states during walking and running (30 steps test for each trial).

	**Number of missing states in walking**			**Number of missing states in running**			**Missing probability [%]**
	**1st**	**2nd**	**3rd**	**1st**	**2nd**	**3rd**	

**State 1**	0	0	0	20	28	14	34.4
**State 2**	0	0	0	0	0	0	0.0
**State 3**	0	0	0	0	0	0	0.0
**State 4**	0	0	0	13	14	3	16.7

**Table 2.** Number of detected zero velocity intervals by different methods (actual number of zero velocity interval is 30).

	**Experiments**	**Speed [m/s]**	**Number of detected zero velocity intervals by different methods**				
			**[1]**	**[2]**	**[3]**	**Proposed method with an HMM filter**	**Proposed method with an HMM smoother**

**Walking**	1st	1.3	28	30	30	30	30
	2nd	1.4	22	29	30	30	30
	3rd	1.4	28	30	30	30	30

**Running**	1st	2.4	0	2	30	10	30
	2nd	2.8	0	1	30	1	30
	3rd	2.5	0	0	30	16	30

**Table 3.** Number of falsely detected zero velocity intervals by different methods.

	**Experiments**	**Speed [m/s]**	**Number of detected non zero velocity intervals by different methods**				
			**[1]**	**[2]**	**[3]**	**Proposed method with an HMM filter**	**Proposed method with an HMM smoother**

**Walking**	1st	1.3	0	0	5	0	0
	2nd	1.4	0	0	1	0	0
	3rd	1.4	0	0	1	0	0

**Running**	1st	2.4	0	0	0	0	0
	2nd	2.8	0	0	0	0	0
	3rd	2.5	0	0	3	0	0
